# Midwives’ perspectives on assessing and managing mothers’ distress related to excessive infant crying in Japan: a qualitative content analysis study

**DOI:** 10.1186/s12884-025-08278-5

**Published:** 2025-12-29

**Authors:** Sayo Kikuchi, Toshiko Himeno

**Affiliations:** 1 Japanese Red Cross Kyushu International College of Nursing, 1-1 Asty Munakata-shi, Fukuoka, 811-4157 Japan; 2 Doctoral Program, Graduate School, Department of Reproductive Health Nursing, Japanese Red Cross Kyushu International College of Nursing, 1-1 Asty, Munakata-shi, Fukuoka, 811-4157 Japan

**Keywords:** Excessive crying, Infant crying, Midwifery, Postpartum care, Qualitative research

## Abstract

**Background:**

Excessive infant crying poses significant challenges to maternal well-being, often leading to parenting stress and postpartum depression. Midwifery assessment and support for mothers experiencing this distress remain inconsistent, emphasizing the need for evidence-based interventions in postpartum care.

**Methods:**

This qualitative descriptive study recruited five midwives in Japan using a combination of purposive and snowball sampling. Semi-structured interviews were conducted between August and November 2023 to explore their perspectives on assessing and managing maternal distress related to excessive infant crying. Interview transcripts were analyzed using inductive content analysis to identify key assessment categories and nursing care approaches.

**Results:**

Seven assessment categories emerged: (1) Maternal psychological burden, (2) Maternal physical burden, (3) Maternal engagement with excessive crying, (4) Infant physical factors related to excessive crying, (5) Infant sleep disturbances related to irregular routines, (6) Characteristics of crying, and (7) Breastfeeding-related issues. The findings illustrate that midwives provided holistic support by addressing both maternal and infant needs. Midwives offered psychological support to alleviate mothers’ self-blame, promoted family involvement, and provided practical guidance on infant care, such as touch-based interventions and regulating sleep-wake cycles. While all participants acknowledged the necessity of a comprehensive approach, the primary focus of their interventions varied.

**Conclusions:**

This study highlights the role of midwives in identifying and addressing maternal distress during the postpartum period. The findings suggest that excessive crying often stems from an intricate interplay between maternal psychological burdens and infant-specific factors. However, the varied emphasis of interventions underscores the need for structured, evidence-informed midwifery practices to ensure consistent care. Furthermore, this study suggests that many mothers in Japan tend to blame themselves when their infant cries, a reaction that may arise in other cultural contexts. Greater insight into how maternal self-perceptions shape coping strategies across cultures could help develop more effective postpartum care worldwide.

**Supplementary Information:**

The online version contains supplementary material available at 10.1186/s12884-025-08278-5.

## Background

 Infant crying constitutes a fundamental means of communication, enabling newborns to convey their physiological and psychological needs to caregivers [1]. This reciprocal interaction facilitates parent-infant bonding as caregivers gradually learn to interpret and respond to their infant’s cues [2]. Typically, infant crying follows a developmental trajectory, increasing around two weeks of age, peaking at approximately five to six weeks, and declining by four to five months [3, 4]. However, some infants display prolonged and excessive crying that surpasses typical developmental patterns, a condition clinically referred to as “infantile colic.” According to the Rome IV criteria, colic is defined as recurrent and extended crying, fussing, or irritability in otherwise healthy infants under five months, occurring without identifiable cause or effective soothing methods [5]. Globally, colic affects about 10–15% of infants [6], and 9.3% of Japanese infants reportedly experience this condition [7], a rate that is lower than in many Western countries [8]. Such persistent fussing remains one of early infancy’s most frequently cited parental concerns [9].

Excessive infant crying can have a profound impact on maternal well-being, leading to physical fatigue, heightened parenting stress, and postpartum depression [10, 11]. In Japan, the prevalence of postpartum depression at one month after childbirth is reported to be 14.3% [12], underscoring the significant psychological challenges that mothers face during the early postpartum period. Alongside this, maternal distress related to excessive infant crying tends to peak at one month postpartum [13], precisely when midwives take on a pivotal role in providing postpartum care and support for mothers and infants. Although crying-related distress is widely recognized, its assessment and management are not comprehensively standardized in midwifery practice.

In addition, excessive crying has been identified as a key risk factor for shaken baby syndrome (SBS) and other forms of child maltreatment [14]. One study in Japan showed that 3.9% of mothers admitted to shaking their infants at least once out of frustration with ongoing crying [15], illustrating the urgent need for early intervention and parental support strategies. Efforts to mitigate these risks have included various international programs. For instance, the Neuroprotective Developmental Care (NDC) approach combines infant behavioral insights with parental support to lower excessive crying and improve maternal well-being [16]. Yet in Japan, national postpartum care programs predominantly center on maternal physical recovery and breastfeeding support, commonly overlooking the psychological burden of managing infant crying. Due to the lack of standardized guidelines [17], many midwives rely on personal experience when advising mothers, although efforts to establish evidence-based recommendations are underway [18]. Thereby underscoring the need for more formalized, midwifery-led interventions to ensure holistic postpartum care.

Although medical and behavioral strategies have played a central role in addressing excessive infant crying [19], they often leave the mother’s emotional distress insufficiently resolved. Midwives, serving as frontline caregivers in the postpartum period, are essential for assisting mothers grappling with persistent crying but often focus on infant-related factors to the detriment of maternal psychological well-being. Educational interventions aimed at improving maternal knowledge have demonstrated benefits, yet they can lack personalized emotional support [20]. In clinical practice, many midwives turn to their own experience rather than standardized, evidence-based approaches, resulting in inconsistent care delivery. Without structured guidelines, the effectiveness of midwifery-led interventions remains uncertain, and the support offered to distressed mothers varies widely. These limitations highlight the need for comprehensive, midwifery-led strategies that integrate practical and emotional assistance for mothers, alongside establishing standardized assessment tools and protocols to enable consistent, high-quality postpartum care.

Despite the well-documented repercussions of excessive infant crying on maternal well-being, a significant gap remains in understanding how midwives currently assess and address crying-related distress in mothers. Midwives frequently function as the earliest professional point of contact for maternal concerns, offering direction on infant behavior and maternal coping. Yet the scope and consistency of those assessments vary across clinical settings, given that many midwives rely on experiential knowledge instead of standardized protocols.

This study aims to fill this gap by examining midwives’ perspectives on and practices for assessing and managing maternal distress linked to excessive infant crying, using a qualitative approach based on semi-structured interviews. By studying midwives’ lived experiences, the research seeks to pinpoint principal challenges and best-practice strategies that can strengthen midwifery care globally. The findings will create structured assessment frameworks and foster more systematic, effective midwifery-led interventions. Beyond these clinical implications, this study is significant for midwifery education and professional development, supporting midwives’ central position in addressing infant crying-related distress and promoting maternal well-being in the postpartum period worldwide.

## Methods

### Study design

This study employed a qualitative descriptive design to explore how midwives in Japan assess and manage maternal distress associated with excessive infant crying. Qualitative methodology was chosen to capture the nuanced perspectives of midwives on both the mothers’ backgrounds and the specific nursing interventions used to address excessive crying.

### Definition of “Excessive Crying”

In this study, “excessive crying” was defined by referencing the clinical perspective of the Rome IV criteria for infantile colic [5], which emphasizes caregiver perception. Accordingly, we defined it as any crying that mothers perceive as prolonged and unsoothable, which becomes a source of their distress.

### Recruitment of participants

Participants were midwives providing nursing care for mothers coping with challenging infant crying in neonatal home visits or postpartum care services. To ensure sufficient professional expertise, the Japanese Midwifery Clinical Ladder served as a reference, and midwives with at least six years of experience beyond the essential competency acquisition phase were targeted.

Recruitment was conducted using a combination of purposive and snowball sampling. Initially, the researcher purposively invited two midwives known to her who met the inclusion criteria. Subsequently, a professional contact (also a midwife) was asked to identify and refer other midwives within her professional network who also met the selection criteria. This snowball sampling approach yielded three additional participants, resulting in a final sample of five midwives, all of whom completed the study. Recruitment was concluded with five participants because thematic saturation was reached, as no new significant themes emerged in the fifth interview.

### Data collection

From August to November 2023, data were collected by the first author via semi-structured interviews, each lasting approximately 60 min. Depending on participant availability, one-on-one interviews took place face-to-face or via an online platform. The interviews followed a protocol covering two main topics: (1) assessment perspectives used by midwives to determine relevant background and observational criteria for mothers and infants, and (2) the nursing interventions implemented in practice, including practical guidance, methods of engaging with distressed mothers, and strategies for emotional support. All interviews were recorded with participant consent, and verbatim transcripts were subsequently prepared for analysis. The interview guide used in this study is available as Supplementary File 1.

### Data analysis

An inductive content analysis was conducted to systematically identify midwives’ assessment approaches and nursing practices. No qualitative data analysis software (e.g., NVivo) was used for this study; the analysis was conducted manually.

First, each recorded interview was transcribed verbatim and scrutinized to extract meaning units related to assessment criteria and intervention methods. These meaning units were then highlighted and assigned a code while preserving contextual integrity. These initial codes were grouped into subcategories based on semantic commonalities, which were further consolidated into broader conceptual categories. Examples of the coding process are provided in Supplementary File 2. In the final stage, relationships between assessment items and nursing interventions were examined to establish a structured understanding of midwifery care in managing excessive infant crying.

To ensure analytical rigor, the analysis was conducted collaboratively by two researchers: the first author (a researcher with 18 years of clinical midwifery experience) and the co-author (a senior researcher with extensive experience in qualitative nursing research). The first author performed the initial coding and categorization. Following this, both authors engaged in thorough discussions to review all codes and categories. Any discrepancies in interpretation were resolved by repeatedly returning to the verbatim transcripts and discussing the findings until a consensus was reached. This process aimed to mitigate potential biases and ensure that the findings accurately represented midwives’ practices.

### Rigor and trustworthiness

Several strategies were employed to ensure the rigor and trustworthiness of this study. The first author (MHS), a female researcher with 18 years of clinical experience as a midwife, brought deep contextual understanding to the data. To manage the potential bias arising from this background, a reflexive approach was maintained throughout the research process. The collaborative analysis with the co-author (PhD), who has extensive experience in supervising qualitative nursing research, served as peer debriefing. Through regular discussions, interpretations were continuously challenged to ensure they were grounded in the participants’ accounts rather than the researcher’s preconceptions.

To further enhance the credibility and dependability of the findings, member checking was conducted. After the initial analysis, the researchers presented each participant with their verbatim quotes aligned with the generated codes, allowing them to confirm that the interpretations accurately reflected their intended meanings. Based on their feedback, minor modifications were made to the wording of some codes to ensure precision. An audit trail, consisting of verbatim transcripts, coding notes, and records of category development, was also meticulously maintained.

To ensure the semantic accuracy of participant quotes translated from Japanese, a two-step process was employed. First, the initial translation was performed by a professional editor specializing in academic nursing manuscripts. Second, the first author, as the content expert, meticulously reviewed the translated text against the original transcripts to confirm that all clinical nuances and intended meanings were faithfully preserved.

Finally, this study’s reporting adheres to the Consolidated criteria for reporting qualitative research (COREQ) guidelines (Supplementary File 3).

### Ethical considerations

This study was approved by the Research Ethics Committee of the Japanese Red Cross Kyushu International College of Nursing (Approval No. 23 − 014) and conducted following the Declaration of Helsinki. Before each interview, participants were informed of the study’s aims, procedures, potential risks and benefits, and their voluntary right to withdraw without penalty. They were also assured they could avoid answering any questions they found uncomfortable. Written informed consent was obtained from all participants before the interviews commenced. Identifying details (e.g., personal names, locations, or sensitive information) were removed or replaced with unique identifiers to protect anonymity. All collected data were securely stored under these assigned codes, and only the research team had access to the linkage between participant identities and their transcripts.

## Results

### Participant characteristics

**Fig. 1:**
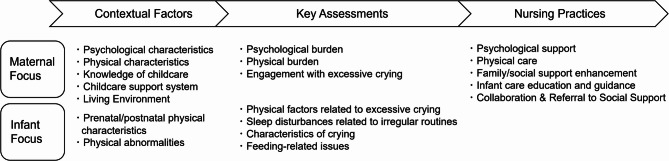
A framework of midwifery care for mothers distressed by excessive infant crying

Five midwives with 12 to 46 years of experience participated in this study (Table 1). The analysis of the interviews revealed a comprehensive process of midwifery care. To visually summarize our findings, we developed “A Framework of Midwifery Care for Mothers Distressed by Excessive Infant Crying” (Fig. 1).

**Table 1:** Characteristics of midwife participants (*N* = 5)

	MW1	MW2	MW3	MW4	MW5
Age	60s	30s	50s	50s	30s
Midwife Experience (years)	46	13	17	29	12
EC Support Experience (years)*	33	8	2.5	17	11
Region^†^	A	B	C	A	A
Position					
Director of a Midwifery Clinic		〇	〇	〇	〇
Municipal Government-appointed Midwife	〇	〇			
Part-time Instructor at a Midwifery School					〇
Services Provided					
Postpartum Care Program					
Daycare			〇	〇	〇
Outreach		〇			
Short-Stay			〇		
Neonatal Home Visiting Program	〇	〇	〇		
Parenting Support Services				〇	〇

*EC Support Experience refers to the number of years of experience in providing support for excessive crying

†Regions A, B, and C represent three different geographical areas in Japan

This framework illustrates the entire care process, from the assessment of contextual and current factors to the implementation of integrated nursing practices. A summary of the key assessment categories and nursing practices is presented in Table 2, with the full detailed tables available in Supplementary Files 5 and 6. The detailed table for the contextual factors is provided in Supplementary File 4. The following sections explain the key findings along this framework.

**Table 2:** Summary of midwives’ assessment categories and nursing practices

Theme/Category	Subcategory	Representative Assessment	Representative Nursing Practice
**Theme 1: Maternal Assessment and Nursing Practices**			
Psychological Burden	Parenting Stress	Accumulated stress from childcare	Use empathic communication to reduce maternal tension
	Self-blame in Childrearing	Self-blame related to the infant’s crying	Emphasize that the infant’s refusal is not directed against the mother
	Anxiety about Childrearing	Uncertainty about the cause of crying	Explain typical crying phases and their relation to development
	Negative Emotions toward Birth Experience	Lingering emotional distress about the childbirth experience	Listen empathetically to the mother’s birth story
	Excessive Focus on the Infant	Mother is overly focused on the infant, making it difficult to remain calm	Encourage the mother to occasionally set the infant down so she can gain perspective
	Psychological Burden from Relationship with Own Mother	Mother’s self-efficacy declines due to her own mother’s imposed childcare beliefs	Promote interaction with mothers of the same generation to share childrearing perspectives
	Need for Ongoing Support for Mother and Child	Mother’s difficulty in childcare is severe and not resolved by one-time support	Establish follow-up plans and refer to community or specialized services as needed
Physical Burden	Degree of Fatigue	Level of fatigue	If fatigue is severe, suggest using services such as postpartum daycare to rest
	Sufficiency of Meals and Sleep	Inadequate food or sleep due to constant crying management	Propose ways to utilize community resources for short-term breaks
	Lifestyle Constraints Caused by Crying Management	Mother delays her meals or bathroom breaks to address crying	Guide the mother to prioritize her basic needs, such as meals and bathroom breaks, to enable self-care
	Muscle Tension around the Scapula Due to Breastfeeding	Shoulder blade stiffness caused by breastfeeding posture	Teach exercises to relax the scapular region
Engagement with Excessive Crying	Reaction to Excessive Crying	Overly sensitive response to crying	Validate the mother’s responses and provide concrete guidance on interpreting and managing crying
	Perceptions of Crying	How the mother interprets the infant’s crying	Reinforce that crying serves as a communicative signal, encouraging more objective thinking
	Interaction with the Infant	Lack of familiarity with physical touch or verbal engagement	Provide a model by carefully showing how to hold and touch the infant
**Theme 2: Excessive Infant Crying Assessment and Nursing Practices**			
Physical Factors related to Excessive Crying	Degree of Muscle Tension	Evaluate the infant’s muscle tension (e.g., observing the infant in a prone position)	Teach the mother how to use touch to relieve muscle tension
	Head-turning Preference	Check for any consistent preference for turning the head to one side	Provide playful parent-child exercises to correct lateral differences
	Holding Methods	Observe the holding posture	Teach methods to approximate the fetal-like position while holding
	Hypersensitivity to External Stimuli	Check if the Moro reflex occurs frequently	Use baby massage or skin-to-skin contact to reduce hypersensitivity
	Presence of Physical Abnormalities	Check for any physical abnormalities in the infant	Encourage a pediatric consultation if any physical abnormalities are suspected
	Growth and Development	Observe any recent rapid growth or developmental changes	Explain that crying may temporarily increase during periods of rapid development and reassure the mother
Sleep Disturbances Related to Irregular Routines	Irregular Daily Rhythms	Record bedtime, wake-up time, and morning/evening naps	Recommend a morning walk, reducing activity in the evening to aid nighttime sleep
	Poor Sleep	Assess whether the mother recognizes signs of infant drowsiness	Explain how to detect sleep cues (e.g., rubbing eyes, fussing) and transition the infant to sleep
Characteristics of Crying	Cry Presentation	Note timing and duration of crying	For crying that escalates toward evening, suggest reviewing the infant’s daytime sleep and level of activity
	Factors Associated with Crying	Determine whether crying increases concurrently with sudden growth or developmental milestones	Inform the mother that transient increases in crying often occur around 3 weeks, 3 months, 5 months, and 6 months
Feeding-related Issues	Relation Between Crying and Feeding	Check if the mother is feeding very frequently just to stop the crying	Brainstorm alternate methods of soothing (e.g., holding, play) aside from feeding
	Feeding Problems	Check if the infant has difficulty sucking or if the mother is inexperienced in breastfeeding	Offer detailed instruction on feeding positions and techniques

### Assessment items for maternal and child background

Before proposing interventions, each midwife emphasized a comprehensive review of maternal and infant factors (Table 2). On the maternal side, this included investigating anxiety or depression, cumulative stress, and any physical difficulties such as postpartum fatigue. Participants explained that heightened distress often arose from insufficient support or an inability to rest and recover physically. MW3, who regularly encountered mothers reporting acute “childcare fatigue,” commented on the interplay of inadequate assistance and difficulty managing an infant’s crying:

“They come in describing it as ‘childcare fatigue,’ but once I look at the details, it often involves a lack of support or inability to handle the baby’s needs. That leads to emotional exhaustion, plus they can’t sleep and become physically overwhelmed, so in the end, both mind and body tend to collapse.” (MW3).

Infant-specific aspects such as muscle tension, feeding habits, and prenatal/perinatal factors were also evaluated. Two midwives described cases in which strong arching or signs of discomfort correlated with vacuum extraction at birth or maternal back pain during pregnancy. In line with previous findings, participants underscored how these background elements shaped their guidance regarding feeding frequency, handling techniques, and daily infant routines.

### Maternal assessment and nursing practices

Participants typically implemented maternal-oriented measures once assessments were completed, as summarized in Table 2. Addressing maternal stress was a recurring priority. MW1 recalled that mothers who were incredibly earnest or perfectionistic in childcare frequently perceived moderate crying as extreme, remarking:

“Mothers who have accumulated considerable stress, or are earnest and highly dedicated to childrearing, … report that their baby ‘cries a lot’ even if it’s only a small amount of crying.” (MW1).

This mindset could exacerbate anxiety and lead to overreactions, potentially heightening tension around the baby.

MW2 observed that many mothers openly blamed themselves when failing to soothe an infant promptly:

“They feel like ‘I’m the one at fault, I must be inadequate,’ and I get the impression that a lot of them are blaming themselves.” (MW2).

Midwives described counteracting these tendencies by reframing crying as a normal communicative behavior and explicitly acknowledging a mother’s efforts. MW3 noted how readily some mothers attributed prolonged fussiness entirely to their own perceived shortcomings:

“Surprisingly, there are quite a few mothers who regard everything as their fault.” (MW3).

Furthermore, midwives noted how distress can be compounded by both external social pressures and an intense internal focus. MW5 described this dual burden:

“Mothers worry… for example, if it’s an apartment with thin walls, they are concerned about bothering the neighbors… [On top of that,] when the baby is crying, they stop even thinking about going out. They’re just desperate to make the crying stop.” (MW5).

Participants found empathic listening and gentle reassurance helpful in alleviating such self-blame. They also focused on practical ways to reduce physical strain or fatigue, since many mothers struggled with interrupted sleep, minimal meal breaks, and persistent guilt about letting the baby cry for even brief intervals.

### Excessive infant crying assessment and nursing practices

All participants highlighted the importance of carefully observing the baby’s physiological cues (see Supplementary Files 5 and 6 for the detailed tables). MW3 mentioned that mothers who sought postpartum care often arrived complaining of “too much crying” but that the deeper issue lay in how they perceived crying and attempted to eliminate it at all costs:

“There’s a lot of crying… They’re wearing themselves out by trying too hard to make the baby stop crying. Ultimately, I think the real issue is less about the baby itself and more about how the mother or family views the situation.” (MW3).

Such insights led midwives to concentrate on whether the baby was overtired or hungry and whether the mother or family felt pressured to prevent any crying altogether. Training mothers to interpret “fussing” differently, establishing consistent naps or bedtimes, and coaching on gentle handling or swaddling were commonly reported strategies. Midwives explained that ensuring sufficient daytime activity was crucial for promoting better nighttime sleep. MW4 described her specific advice on structuring the infant’s day:

“To help them sleep well at night, the amount of activity during the day becomes important. So, I advise them to go for a walk outside in the morning… and to switch to quiet play in the evening that isn’t too exciting.” (MW4).

Separately, overfeeding was another concern if mothers believed every cry indicated hunger, so midwives tried to help mothers distinguish feeding cues from other forms of restlessness.

### Challenges and future recommendations

Despite reporting effective interventions, participants mentioned challenges. Some mothers, especially those lacking family help, found it hard to sustain recommended practices over time due to persistent exhaustion. Others hesitated to allow an infant even short periods of fussing, undermining strategies aimed at guided self-soothing or differentiating cry patterns. Participants considered repeated follow-up care or online check-ins crucial since a single visit rarely sufficed to ease anxiety or correct ingrained misinterpretations about crying. They also urged broader awareness that crying does not equate with substandard parenting. As MW3 summarized,

“I often start by explaining that they shouldn’t assume from the outset that ‘you must never let the baby cry.’ It seems many mothers regard the fact that a baby is crying as a sign of poor parenting.” (MW3).

Overall, the midwives advocated for a multifaceted approach: expanding postpartum day-care programs for maternal rest, fostering supportive home environments, and reinforcing that infant crying is natural and not invariably the mother’s fault. They saw promise in consistent postpartum guidance, a combination of in-person and digital resources, and the empowerment of mothers to accept brief fussiness as part of early infancy.

## Discussion

This study revealed that midwives offered various nursing practices based on a multifaceted assessment of both mothers and infants while providing support for mothers distressed by their infants’ excessive crying. The findings underscore that infant crying is not solely driven by “physical tension” in the infant but also by the mother’s condition, including her stress levels, breathing patterns, daily rhythms, and psychological well-being. Consequently, an approach that goes beyond simply “stopping the infant from crying” is required, favoring instead the establishment of a comprehensive foundation of environment, routine, and physical contact that collectively help reduce crying episodes.

### The importance of comprehensive assessment and psychological support for mothers

The results confirmed that midwives’ assessments extended beyond practical or technical considerations of handling excessive infant crying. They also encompassed mothers’ psychological burdens, such as self-blame, anxiety, along with social factors like the level of family support. Our findings suggest that mothers with insufficient partner or family support are particularly prone to isolation and fatigue, which further intensifies their distress.

These elements align with prior research suggesting that prolonged infant crying triggers maternal distress and exacerbates parenting anxiety if not promptly addressed [21]. This tendency appears particularly pronounced due to the cultural and social background in Japan. Chen & Miyake [22] point out that in Japanese child-rearing, negative infant states such as crying are often regarded as the mother’s responsibility, and that mothers strive to avoid crying in order not to be criticized by neighbors. This sense of a heavy responsibility is so profound that, as our study also found, mothers often put off their own eating and sleeping to respond to their infant’s crying, sometimes worsening their stress to the point of physical and emotional collapse. This cultural pressure, which frames crying as a sign of maternal failure, is a major factor driving mothers to self-blame and intense anxiety.

Another key theme was how a mother’s perspective on crying can drive a vicious cycle of guilt and stress. This is a critical process, as a mother’s perception of crying has been shown to influence her attachment and subsequent parenting stress [23], while her beliefs about infant crying are themselves shaped over time by the infant’s temperament [24]. This dynamic presents a clinical challenge, as the resulting shift toward more parent-oriented beliefs can be an adaptive response for the mother, yet al.so a long-term behavioral risk for the child.

Therefore, the midwives’ role in reframing crying from a sign of maternal failure to a normal communicative behavior is pivotal. This intervention is a vital support for both mother and child; helping the mother adapt psychologically while mitigating developmental risks. Consistent with this approach, broader studies show that programs supporting mothers’ psychological and physical states can reduce anxiety around crying [25, 26], and recent findings also indicate that maternal psychological well-being can be a protective factor against infant colic [27]. This comprehensive assessment of the mother’s daily circumstances suggests that midwives view these various factors not in isolation, but as being deeply intertwined with the mother’s psychological state.

### Focusing on the physical factors surrounding excessive infant crying

Midwives’ careful evaluation of infant muscle tension, habitual head-turning, and other physical attributes underscores the importance of assessing “why the baby cries” from a physiological standpoint. High muscle tone or significant arching often correlates with irritability and persistent crying [28, 29], suggesting that physical factors may need greater scrutiny if neither hunger nor a soiled diaper explains the fuss.

However, a recent systematic review concluded that certain complementary and alternative approaches may not significantly reduce crying duration in colicky infants [30]. This aligns with the practices observed in our study, where midwives employed methods such as gentle tactile care rather than more invasive manual therapies, suggesting an implicit understanding of the need for a cautious, evidence-informed approach. The participants in this study guided mothers in employing swaddling, gentle tactile care, and appropriate positioning to ease tension, thus facilitating calmer feeding and more settled periods of rest.

This focus on a “physical approach” complements maternal psychological support. However, this also highlights the complexity and need for caution with such interventions. For instance, some research indicates that while a manual therapy like chiropractic care may not show a significant effect on average, there could be subgroups of infants, such as those with musculoskeletal problems, who benefit more [29], underscoring the limited evidence base. Similarly, another study found that the effectiveness of swaddling was dependent on the infant’s age [31]. These findings demonstrate that such physical interventions must be applied not only with individualized assessment but also with strict adherence to safety guidelines. For example, the American Academy of Pediatrics recommends discontinuing swaddling once an infant shows signs of rolling to prevent sleep-related deaths [32]. This underscores the importance of the individualized, evidence-informed, and safety-focused assessments observed in our study, rather than applying a one-size-fits-all approach.

In line with the bullet point emphasizing the influence of pregnancy and birth history, this study found that vacuum extraction or maternal posture during pregnancy could predispose infants to muscle tension, increasing fussiness post-birth. Addressing such factors might entail adjusting daily rhythms, for example, advising mothers to establish morning walks or earlier bedtimes that benefit both the mother’s recovery and the infant’s physiological regulation [33]. The results thus point to a broader emphasis on “foundational approaches,” including environment adjustments, daily routines, and gentle physical contact, that aim to prevent crying from escalating in the first place.

### Issues and prospects for individualized care encompassing mothers and infants

The findings suggest a cyclical relationship in which maternal anxiety, guilt, and lack of support interact with an infant’s muscle tension or discomfort, exacerbating crying durations. Conversely, the burden on the mother may be reduced by integrated approaches addressing her emotional state and the infant’s physical condition, as indicated by M O’Higgins, I St James Roberts and V Glover [26]. Such integrative strategies are crucial not only to forestall postpartum depression or childcare anxiety but also to lower the risk of abusive incidents triggered by incessant crying.

However, participants emphasized the necessity for multidisciplinary collaboration and greater use of social resources. Midwives alone may be overextended if tasked with handling maternal mental health, infant muscle tension, daily routine adjustments, and broader family dynamics. Collaboration with psychiatrists, counselors, child welfare agencies, and community-based programs can help address the multiple domains affecting maternal and infant well-being [29, 34]. In line with the suggestion that experiences during pregnancy or childbirth and the presence of robust family support can be crucial, it may prove beneficial to detect potential risks for excessive crying as early as pregnancy. Comprehensive postpartum planning could then be tailored to each mother-infant dyad.

Moreover, while some research has explored manual therapies or structured interventions for excessive crying, a meta-analysis indicated these may not consistently benefit mother–infant outcomes [35]. This study highlights practical recommendations: first, the development of integrated support models that simultaneously evaluate maternal stressors, infant tension, and family involvement; second, the creation of tools that combine psychological and physical assessments in standardized midwifery-led interventions; and third, establishing follow-up frameworks to continue individualized interventions beyond a single home visit.

To enhance the continuity and comprehensiveness of care, a structured follow-up model could be highly effective. Such a model might include an initial in-person postpartum consultation, followed by a telehealth or phone check-in after one to two weeks to assess progress. If concerns persist, an in-person reassessment could be scheduled, alongside establishing a clear pathway for referral to appropriate specialized support services when necessary. Taken together, these measures would not only help in managing specific issues or persistent crying, but would also provide mothers with sustainable and safe alternatives to constant feeding or holding, thereby fostering maternal confidence and well-being.

### Limitations

Several limitations of this study should be acknowledged. First, the small sample size (*N* = 5) means that the findings cannot be generalized to all midwives in Japan. While we recruited participants from three different geographical regions (represented as Regions A, B, and C) to ensure some contextual variation, the sample was drawn from a limited number of areas, and the findings may not be transferable to all clinical settings. Second, the use of purposive and snowball sampling may have introduced selection bias, as participants were recruited from existing professional networks, and may have led to social desirability bias in their responses. Third, this study captured the perspectives of midwives only. The actual experiences of mothers coping with excessive infant crying may differ from the midwives’ descriptions.

### Directions for future research

Building on the limitations of this study, several avenues for future research are recommended. Future studies should aim to recruit a larger and more diverse sample of midwives from a wider range of clinical settings and geographical regions. It would also be invaluable to incorporate the perspectives of mothers, fathers, and grandparents to gain a multi-faceted understanding of crying-related distress. Quantitative or mixed-methods studies could effectively evaluate the efficacy of specific physical interventions, such as swaddling or gentle tactile care, and examine the influence of community-level resources on parental well-being. Furthermore, longitudinal research that follows families from pregnancy through the postpartum period could illuminate how early planning and continuous midwifery support can mitigate crying-related distress and strengthen the evidence base for the formulation of definitive clinical guidelines.

## Conclusion

This study suggests that excessive crying often stems from an intricate interplay between maternal psychological burdens—such as guilt and anxiety—and infant-specific factors, including heightened muscle tension and disrupted daily rhythms. The findings indicate that comprehensive midwifery-led interventions addressing both maternal mental health and infant physical care are vital for alleviating crying-related distress. This study highlights the role of midwives in identifying and addressing maternal distress during the postpartum period and underscores the need for structured, safety-focused, and evidence-informed midwifery practices.

## Supplementary Information

Supplementary Material 1: Supplementary file1. Interview guide.

Supplementary Material 2: Supplementary File 2. Examples of the inductive content analysis process.

Supplementary Material 3: Supplementary File 3. COREQ (Consolidated criteria for reporting qualitative research) Checklist.

Supplementary Material 4: Supplementary File 4. Detailed assessment items for maternal and infant contextual factors.

Supplementary Material 5: Supplementary File 5. Detailed maternal assessment and nursing practices

Supplementary Material 6: Supplementary File 6: Detailed excessive infant crying assessment and nursing practices.

## Data Availability

The datasets generated and/or analyzed during the current study are available from the corresponding author upon reasonable request. To protect participant privacy, all de-identified data are stored on secure, access-controlled institutional drives for 10 years and are accessible only to the research team. All supplementary materials referenced in this manuscript—including the interview guide (Supplementary File 1), examples of the inductive content analysis process (Supplementary File 2), the COREQ checklist (Supplementary File 3), and detailed results tables (Supplementary Files 4–6)—are provided with this submission.
