# Supplementary material for: Midwives’ perspectives on assessing and managing mothers’ distress related to excessive infant crying in Japan: a qualitative content analysis study
Source: BMC Pregnancy Childbirth. 2025 Dec 29;25:1324. doi: 10.1186/s12884-025-08278-5 (PMC12752126; doi:10.1186/s12884-025-08278-5)
Supplement: Supplementary file 1 — Supplementary Material 1: Supplementary file1. Interview guide. [file 12884_2025_8278_MOESM1_ESM.docx]

**Supplementary file1: Interview guide**

Interview Questions:

・Could you describe the support you provide to mothers struggling with excessively crying infants? Please share your experiences.

I will further explore the following aspects based on the responses of the participants:

・What information do you gather when assessing these situations? (e.g., mother-infant background)

・What do you observe when providing support? (e.g., mother-infant interactions, conditions)

・What specific support measures do you implement? (e.g., guidance, engagement, assistance)
