# Supplementary material for: Midwives’ perspectives on assessing and managing mothers’ distress related to excessive infant crying in Japan: a qualitative content analysis study
Source: BMC Pregnancy Childbirth. 2025 Dec 29;25:1324. doi: 10.1186/s12884-025-08278-5 (PMC12752126; doi:10.1186/s12884-025-08278-5)
Supplement: Supplementary file 2 — Supplementary Material 2: Supplementary File 2. Examples of the inductive content analysis process. [file 12884_2025_8278_MOESM2_ESM.docx]

**Supplementary File 2: Examples of the Inductive Content Analysis Process**

To enhance the transparency of our analysis, this table presents the inductive process through which themes were derived. It shows how verbatim quotes from participants were initially coded, then organized into subcategories, and finally consolidated into broader categories.

| **Verbatim Quote** | **Code** | **Subcategory** | **Category** |
| --- | --- | --- | --- |
| **Theme 1: Maternal Assessment and Nursing Practices** | | | |
| "Ultimately, they come in with what you could broadly call 'childcare fatigue'... They can't sleep and are at their physical limit, so usually, both mind and body collapse." (MW3) | Accumulated stress from childcare | Parenting Stress | Psychological Burden |
| "I get the impression that they feel like, 'It's my fault. It's because I'm bad at this.'" (MW5) | Self-blame related to the infant’s  crying | Self-blame in  Childrearing |  |
| "They're distressed because they don't know why the baby is crying..." (MW5) | Uncertainty about the cause of  crying | Anxiety about  Childrearing |  |
| "In the background of mothers who don't know how to interact with their baby, there are often these lingering, unresolved feelings about the birth experience. So, rather than confirming what happened, it's more about just listening to their story." (MW2) | Lingering emotional distress about the childbirth experience | Negative Emotions  toward Birth Experience |  |
| “When a baby won’t stop crying, it feels like the psychological distance between the mother and baby is too close. I often think the mother becomes overly focused on the infant... and then the anxiety builds, the crying continues, and it just turns into a vicious cycle.” (MW2) | Mother is overly focused on the infant, making it difficult to remain calm | Excessive Focus on  the Infant |  |
| "The generations are a bit different, and they get their own mother's advice pushed on them... some feel like '[my mother] doesn't understand,' or they are told things so forcefully that they start to feel like, 'I'm a bad mother.'" (MW3) | Mother’s self-efficacy declines due to her own mother’s imposed  childcare beliefs | Psychological Burden  from Relationship with  Own Mother |  |
| "If it seems like the issue won't be resolved in a single visit... I might suggest another postpartum care session, a baby massage class, or getting out to a local parenting salon... I try to connect them to someone." (MW5) | Mother’s difficulty in childcare is severe and not resolved by one-time support | Need for Ongoing  Support for Mother and Child |  |
| "Before she knows it, night has come, the long night comes again, morning comes... The mother isn't sleeping and is exhausted." (MW4) | Level of fatigue | Degree of Fatigue | Physical Burden |
| "I check the family's life background, including whether the parents, centering their life on the baby, are not sleeping, not eating, or not getting rest." (MW4) | Inadequate food or sleep due to  constant crying management | Sufficiency of Meals  and Sleep |  |
| "...they can't eat properly because they have to hold the baby... They can't even go to the toilet because the baby is crying, leading to constipation, or they hold their urine for so long they have to run to the bathroom." (MW3) | Mother delays her meals or  bathroom breaks to address crying | Lifestyle Constraints Caused by Crying Management |  |
| "People with shallow breathing often have stiff shoulder blades... and with breastfeeding, you see, they get all rigid and stiff, so it gets even worse." (MW1) | Shoulder blade stiffness caused by breastfeeding posture | Muscle Tension around the Scapula Due to Breastfeeding |  |
| "Their response is fast... It's like, 'Oh no, the baby cried! This is an  emergency! What is it?' They often go over as if there's not a second to spare." (MW3) | Overly sensitive response to crying | Reaction to  Excessive Crying | Engagement with Excessive Crying |
| "Many mothers perceive a crying baby as a sign of bad parenting. They tend to think, 'The baby is crying because I'm not looking after it properly.'" (MW3) | How the mother interprets the  infant’s crying | Perceptions of Crying |  |
| "Just from talking [to their baby], some mothers seem very tense or awkward." (MW5) | Lack of familiarity with physical  touch or verbal engagement | Interaction with the  Infant |  |
| **Theme 2: Excessive Infant Crying Assessment and Nursing Practices** | | | |
| "Because the infant drinks in a state of high muscle tension, they cannot get the necessary amount... they doze off, but because they haven't fed properly, they wake up again, leading to frequent feeding." (MW4) | Determine if feeding difficulties  relate to muscle tension | Degree of  Muscle Tension | Physical Factors related to  Excessive Crying |
| "Babies with a strong head-turning preference also cry a lot." (MW1) | Check for any consistent  preference for turning the head to one side | Head-turning Preference |  |
| "Mothers of babies who cry a lot often hold them vertically even at a young age." (MW5) | Observe the holding posture | Holding Methods |  |
| "Hypersensitive babies have the Moro reflex triggered frequently." (MW1) | Check if the Moro reflex occurs  frequently | Hypersensitivity to  External Stimuli |  |
| "As a fundamental premise... I check if there are truly any abnormalities in the infant." (MW3) | Check for any physical  abnormalities in the infant | Presence of Physical  Abnormalities |  |
| "A baby might cry a lot when they are about to go through a developmental leap, like being close to rolling over or teething." (MW5) | Observe any recent rapid growth or developmental changes | Growth and  Development |  |
| "First, I ask about their daily routine and whether the family's lifestyle is functioning." (MW4) | Record bedtime, wake-up time, and morning/evening naps | Irregular Daily Rhythms | Sleep Disturbances related to Irregular Routines |
| "In my impression, mothers of babies who cry a lot... constantly have this desire to somehow get them to sleep... So I ask them if the infant is getting enough physical activity... whether they're in a situation where they can't sleep because their activity level is too low." (MW4) | Note difficulty falling asleep due to inadequate activity | Poor Sleep |  |
| "He cried continuously for about three hours, not getting weaker, but at the same intensity." (MW5) | Note timing and duration of crying | Cry Presentation | Characteristics of Crying |
| "I check their breathing by listening to their cry. Is it a choked-sounding cry, or a 'sucking-in' cry? A reassuring cry, one with a full exhalation, comes from the belly with an 'Aaaah' sound." (MW4) | Observe if there is insufficient  exhalation when crying | Factors Associated with  Crying |  |
| "[Some mothers] feed the baby every time it cries, leading to an unusually high number of feedings." (MW5) | Check if the mother is feeding very frequently just to stop the crying | Relation Between  Crying and Feeding | Feeding-related Issues |
| "Especially since the COVID-19 pandemic, many mothers come home without even knowing the proper way to breastfeed... The baby wants to drink but can't, so they cry... and the mothers feel, 'My baby can't drink,' or 'It's my fault because I'm not good at this.'" (MW5) | Check if the infant has difficulty sucking or if the mother is  inexperienced in breastfeeding | Feeding Problems |  |
