# Supplementary material for: Midwives’ perspectives on assessing and managing mothers’ distress related to excessive infant crying in Japan: a qualitative content analysis study
Source: BMC Pregnancy Childbirth. 2025 Dec 29;25:1324. doi: 10.1186/s12884-025-08278-5 (PMC12752126; doi:10.1186/s12884-025-08278-5)
Supplement: Supplementary file 4 — Supplementary Material 4: Supplementary File 4. Detailed assessment items for maternal and infant contextual factors. [file 12884_2025_8278_MOESM4_ESM.docx]

**Supplementary File4:**

**Detailed Assessment Items for Maternal and Infant Contextual Factors**

| Category | Examples of Specific Assessment Items |
| --- | --- |
| Maternal Psychological Characteristics | ・Mother’s personality (e.g., nervousness, maternal conscientiousness, anxiety tendencies) (MW1, MW3, MW5) |
|  | ・History of mental health issues (MW1) |
|  | ・Subjective evaluation of childbirth experience (e.g., negative emotions) (MW5) |
|  | ・Emotional responses toward the infant (e.g., aversion, anger) (MW1) |
|  | ・Responses to questionnaires (e.g., “Baby’s Feelings Questionnaire”) (MW2) |
| Maternal Physical Characteristics | ・Parity (primipara vs. multipara) (MW2, MW5) |
|  | ・History of threatened preterm labor (MW2) |
|  | ・Body shape and posture during pregnancy (MW5) |
|  | ・Lifestyle and activity patterns during pregnancy (e.g., work status, bedtime) (MW4) |
| Knowledge of Childcare | ・Prior experience interacting with infants (MW3) |
|  | ・Level of childcare-related knowledge (MW3) |
| Childcare Support System | ・Availability and frequency of support from spouse or extended family (MW1, MW3, MW4) |
|  | ・Accessibility of help-seeking resources (MW1) |
|  | ・Specific support provided for household chores and childcare (MW3) |
| Living Environment | ・Housing conditions (e.g., cramped space, thin walls) (MW5) |
|  | ・Sleeping arrangements among family members (MW5) |
| Prenatal Physical Characteristics | ・Presence or absence of vigorous fetal movements (MW1) |
|  | ・Impact of vacuum extraction, fundal pressure, or labor duration on the infant (MW1, MW2) |
|  | ・Delivery mode (e.g., vaginal delivery, cesarean section, vacuum extraction, fundal pressure) and length of labor (MW4) |
|  | ・Fetal posture/position (including a possible link to infant’s head-turning preferences) (MW2, MW4) |
| Postnatal Physical Characteristics and Abnormalities | ・Weight gain (MW1, MW2, MW3) |
|  | ・Growth and developmental progress (MW3, MW5) |
|  | ・Response to auditory stimuli (MW3) |
|  | ・Reactions to soothing and eye contact (MW5) |
|  | ・Presence or absence of physical abnormalities (MW3) |
