# Supplementary material for: Midwives’ perspectives on assessing and managing mothers’ distress related to excessive infant crying in Japan: a qualitative content analysis study
Source: BMC Pregnancy Childbirth. 2025 Dec 29;25:1324. doi: 10.1186/s12884-025-08278-5 (PMC12752126; doi:10.1186/s12884-025-08278-5)
Supplement: Supplementary file 5 — Supplementary Material 5: Supplementary File 5. Detailed maternal assessment and nursing practices [file 12884_2025_8278_MOESM5_ESM.docx]

**Supplementary File5: Detailed Maternal Assessment and Nursing Practices**

| Category | Subcategory | Examples of Assessment | Examples of Nursing Practice |
| --- | --- | --- | --- |
| Psychological Burden | Parenting Stress | ・Accumulated stress from childcare (MW1, MW3) | ・Use empathic communication to reduce maternal tension (MW1)  ・Listen attentively and help clarify specific maternal concerns (MW5) |
|  |  | ・Confusion about being unable to soothe the infant (MW5) | ・Teach breathing techniques for relaxation (MW1) |
|  |  | ・Desire to escape the situation when crying persists (MW1) | ・Emphasize the need to ensure infant safety when the mother steps away (MW1) |
|  |  | ・Reduced motivation to go out due to infant crying (MW5) | ・Suggest using postpartum care or community resources for a change of pace and respite (MW1, MW5) |
|  | Self-blame in Childrearing | ・Self-critical emotions regarding childcare (MW2, MW4) | ・Maintain a non-judgmental attitude (MW2) |
|  |  | ・Belief that she is “not adequately performing” childrearing (MW3) | ・Emphasize that the infant’s refusal is not directed against the mother (MW2) |
|  |  | ・Self-blame related to the infant’s crying (MW3, MW5) | ・Alleviate guilt about temporarily entrusting infant care to others (MW2) |
|  |  | ・EPDS No.3 (“blaming oneself unnecessarily”) (MW2) | ・Avoid blaming the past; focus on identifying actionable steps for now (MW3, MW4) |
|  |  | ・Feelings of guilt toward the infant (MW5) | ・Acknowledge the mother’s efforts verbally (MW5) |
|  | Anxiety about Childrearing | ・Degree of anxiety regarding childcare (MW3) | ・For high-risk cases with severe anxiety, coordinate continuous support with local agencies (MW3) |
|  |  | ・Worry that the infant’s prolonged crying might persist (MW5) | ・Explain typical crying phases and their relation to development (MW5) |
|  |  | ・Uncertainty about the cause of crying (MW5) | ・Introduce community childcare support services (MW3) |
|  |  | ・Restless movements (e.g., rigid holding, rapid rocking) (MW3) | ・Reassure the mother that constant holding is not mandatory (MW3) |
|  | Negative Emotions Toward Birth Experience | ・Lingering emotional distress about the childbirth experience (MW2) | ・Listen empathetically to the mother’s birth story (MW2) |
|  |  | ・Strong sense of self-denial regarding labor (MW3) | ・Help the mother reflect on her delivery, recognizing her efforts (MW3) |
|  | Excessive Focus on the Infant | ・Mother is overly focused on the infant, making it difficult to remain calm (MW2, MW3) | ・Encourage the mother to occasionally set the infant down so she can gain perspective (MW2) |
|  | Psychological Burden from Relationship with Own Mother | ・Mother is confused when her mother’s advice contradicts midwife instructions (MW3) | ・Encourage seeking childcare advice from peers or professionals (MW3) |
|  |  | ・Mother’s self-efficacy declines due to her own mother’s imposed childcare beliefs (MW3) | ・Promote interaction with mothers of the same generation to share childrearing perspectives (MW3) |
|  | Need for Ongoing Support for Mother and Child | ・Mother’s difficulty in childcare is severe and not resolved by one-time support (MW5) | ・Establish follow-up plans and refer to community or specialized services as needed (MW5) |
|  |  | ・Long-term, continuous involvement is needed for mother and infant (MW5) | ・Encourage participation in local community groups, such as parenting salons or baby massage classes (MW5) |
| Physical Burden | Degree of Fatigue | ・Level of fatigue (MW1, MW2, MW4) | ・If fatigue is severe, suggest using services such as postpartum daycare to rest (MW1) |
|  |  | ・Energy depletion from long hours of holding the infant (MW3) | ・Advise the mother to consult her partner, avoiding taking on all childcare responsibilities alone (MW3) |
|  |  |  | ・Provide information on using baby carriers or slings to reduce physical strain (MW3) |
|  | Sufficiency of Meals and Sleep | ・Inadequate food or sleep due to constant crying management (MW2, MW3, MW4) | ・Propose ways to utilize community resources for short-term breaks (MW2) |
|  |  | ・Mother prioritizes childcare over her rest (MW4) | ・Suggest postpartum daycare or other refreshing opportunities (MW4) |
|  | Lifestyle Constraints Caused by Crying Management | ・Mother delays her meals or bathroom breaks to address crying (MW3) | ・Guide the mother to prioritize her basic needs, such as meals and bathroom breaks, to enable self-care (MW3) |
|  |  | ・Fragmented sleep disrupts her daily routine (MW3) | ・Collaborate with the mother to find feasible actions suited to her circumstances (MW3) |
|  | Muscle Tension Around the Scapula Due to Breastfeeding | ・Shoulder blade stiffness caused by breastfeeding posture (MW1) | ・Teach exercises to relax the scapular region (MW1) |
| Engagement with Excsseive Crying | Reaction to Excessive Crying | ・Overly sensitive response to crying (MW1, MW2, MW3, MW5) | ・Validate the mother’s responses and provide concrete guidance on interpreting and managing crying (MW1, MW3) |
|  |  | ・Confusion between fussiness and actual crying (MW1, MW3) |  |
|  | Perceptions of Crying | ・How the mother interprets the infant’s crying (MW3) | ・Reinforce that crying serves as a communicative signal, encouraging more objective thinking (MW3) |
|  | Interaction with the Infant | ・Uncertainty about how to soothe the infant when crying (MW5) | ・Explain the importance of verbal communication with the infant (MW3) |
|  |  | ・Lack of familiarity with physical touch or verbal engagement (MW2, MW3, MW5) | ・If muscle tension is high, demonstrate gentle stroking or touching methods (MW5) |
|  |  | ・Rough handling of the infant (MW2) | ・Provide a model by carefully showing how to hold and touch the infant (MW2) |
