# Supplementary material for: Midwives’ perspectives on assessing and managing mothers’ distress related to excessive infant crying in Japan: a qualitative content analysis study
Source: BMC Pregnancy Childbirth. 2025 Dec 29;25:1324. doi: 10.1186/s12884-025-08278-5 (PMC12752126; doi:10.1186/s12884-025-08278-5)
Supplement: Supplementary file 6 — Supplementary Material 6: Supplementary File 6: Detailed excessive infant crying assessment and nursing practices. [file 12884_2025_8278_MOESM6_ESM.docx]

**Supplementary File6: Detailed Excessive Infant Crying Assessment and Nursing Practices**

| Category | Subcategory | Examples of Assessment | Examples of Nursing Practice |
| --- | --- | --- | --- |
| Physical Factors Related to Excessive Crying | Degree of Muscle Tension | ・Evaluate the infant’s muscle tension (e.g., observing the infant in a prone position) (MW1, MW2, MW4) | ・Gently touch the infant’s head, scapular area, and back (MW1, MW4) |
|  |  | ・Check for arching and its degree (MW1, MW2, MW4) | ・Teach the mother how to use touch to relieve muscle tension (MW2, MW4, MW5) |
|  |  | ・Note difficulty holding the infant, such as the infant’s body not curling easily (MW5) | ・Demonstrate swaddling so the infant’s body can curl more naturally (MW2) |
|  |  | ・Observe trunk, scalp, and neck stiffness (MW3, MW4) | ・Instruct on holding methods that minimize tension (MW4) |
|  |  | ・Determine if constipation or feeding difficulties relate to muscle tension (MW2, MW4) | ・Recommend supervised tummy time, ensuring safety (MW4) |
|  |  | ・Consider if arching leads to difficulty falling asleep (MW4) | ・Supported holding in a fetal-like position to settle the infant (MW2, MW4) |
|  | Head-turning Preference | ・Check for any consistent preference for turning the head to one side (MW1, MW2, MW3) | ・Suggest rearranging the environment to encourage the infant to look toward the less preferred side (MW1) |
|  |  | ・Examine sternocleidomastoid muscle asymmetry and range of visual tracking (MW1, MW4)  ・Observe left-right differences in rooting or sucking reflexes (MW4) | ・Teach light touch care for the tighter sternocleidomastoid muscle (MW1, MW4) |
|  |  | ・Note asymmetry in finger sucking, mouth movement, or eye movement (MW3, MW4) | ・Provide playful parent-child exercises to correct lateral differences (MW1, MW4)  ・Demonstrate proper handling together with the mother (MW3) |

| Physical Factors Related to Excessive Crying | Holding Methods | ・Observe the holding posture (MW1, MW3, MW5) | ・Teach methods to approximate the fetal-like position while holding (MW2, MW3) |
| --- | --- | --- | --- |
|  |  | ・Check whether the infant’s body curls when held (MW5) | ・Explain that this fetal-like position approach is generally recommended until around three months of age (MW3) |
|  |  | ・Note if there is frequent vertical holding in early infancy (MW5) | ・Advise on proper support of the infant’s waist during vertical holding and caution about heat buildup (MW1) |
|  | Hypersensitivity to External Stimuli | ・Determine whether hypersensitivity has been noticeable since birth (MW1) | ・Instruct on swaddling or holding that approximates the fetal-like position (MW1) |
|  |  | ・Check if the Moro reflex occurs frequently (MW1) | ・Demonstrate a bath towel wrapping method usable until about five months of age (MW1) |
|  |  |  | ・Use baby massage or skin-to-skin contact to reduce hypersensitivity (MW1) |
|  | Presence of Physical Abnormalities | ・Check for any physical abnormalities in the infant (MW3) | ・If dietary factors from the mother’s meals might affect the infant, review and offer guidance (MW4) |
|  |  | ・Look for signs of abdominal distension or constipation (MW4) | ・Encourage a pediatric consultation if any physical abnormalities are suspected (MW3) |
|  | Growth and Development | ・Observe any recent rapid growth or developmental changes (MW5) | ・Explain that crying may temporarily increase during periods of rapid development and reassure the mother (MW5) |
| Sleep Disturbances Stemming from Irregular Routines | Irregular Daily Rhythms | ・Record bedtime, wake-up time, and morning/evening naps (MW4, MW5) | ・Encourage setting a consistent wake-up time, aiming for an early-to-bed, early-to-rise habit (MW4, MW5) |
|  |  | ・Check whether a feeding schedule is established (MW4) | ・Suggest adjusting the family’s schedule in tandem with establishing a feeding rhythm (MW4) |
|  |  | ・Note mother’s and father’s daily routines (MW4) | ・Recommend a morning walk, reducing activity in the evening to aid nighttime sleep (MW4) |
|  |  | ・Evaluate the infant’s activity level and timing (MW4)  ・Assess mother’s awareness of her own sleep pattern (MW4) | ・Explain that sleep patterns change as the infant grows (MW4) |
|  | Poor Sleep | ・Identify insufficient daytime sleep (MW5) | ・Advise the mother to let the infant sleep when showing signs of tiredness (MW5) |
|  |  | ・Note difficulty falling asleep due to inadequate activity (MW4) | ・Propose morning walks and midday play to help the infant settle better at night (MW4) |
|  |  | ・Assess whether the mother recognizes signs of infant drowsiness (MW5) | ・Explain how to detect sleep cues (e.g., rubbing eyes, fussing) and transition the infant to sleep (MW5) |
| Characteristics of Crying | Cry Presentation | ・Note timing and duration of crying (MW5) | ・For crying that escalates toward evening, suggest reviewing the infant’s daytime sleep and level of activity (MW5) |
|  |  | ・Check whether crying intensifies in the late afternoon or evening (MW5) |  |
|  |  | ・Observe variations in cry volume and changes (MW5) |  |
|  | Factors Associated with Crying | ・Determine whether crying increases concurrently with sudden growth or developmental milestones (MW5) | ・Inform the mother that transient increases in crying often occur around 3 weeks, 3 months, 5 months, and 6 months (MW5) |
|  |  | ・Observe if there is insufficient exhalation when crying (MW4) | ・If insufficient exhalation is noted, advise taking the infant for a walk to help regulate breathing (MW4) |
| Feeding-related Issues | Relation Between Crying and Feeding | ・Check if the mother is feeding very frequently just to stop the crying (MW4, MW5) | ・Clarify that crying is not always due to hunger (MW5) |
|  |  |  | ・Brainstorm alternate methods of soothing (e.g., holding, play) aside from feeding (MW5) |
|  | Feeding Problems | ・Assess for signs of overfeeding syndrome (e.g., weight gain of ≥50 g/day, abdominal distension) (MW1, MW5) | ・If overfeeding syndrome is suspected, provide feeding guidance (MW1) |
|  |  | ・Consider the possibility of underfeeding (MW4) | ・Offer detailed instruction on feeding positions and techniques (MW5) |
|  |  | ・Check if the infant has difficulty sucking or if the mother is inexperienced in breastfeeding (MW4, MW5) | ・Suggest checking the infant’s sucking ability after relaxing muscle tension (MW4) |
